# Classification of videogames for amblyopia treatment in perceptive and cognitive domains

**DOI:** 10.1371/journal.pone.0335510

**Published:** 2025-10-28

**Authors:** Laura Asensio-Jurado, Marc Argilés, Paula Gil-Llansa, Lluïsa Quevedo-Junyent

**Affiliations:** 1 Centre for Sensors, Instruments and Systems Development (CD6), Universitat Politècnica de Catalunya – BarcelonaTech (UPC), Campus Terrassa, Terrassa, Barcelona, Spain; 2 Facultat d'Òptica i Optometria de Terrassa, Universitat Politècnica de Catalunya – BarcelonaTech (UPC), Campus Terrassa, Terrassa, Barcelona, Spain; 3 Hospital Universitari Mutua Terrassa, Terrassa, Catalunya, Spain; 4 Visió Optometria i Salut, Universitat Politècnica de Catalunya – BarcelonaTech (UPC), Campus Terrassa, Terrassa, Barcelona, Spain; University of Melbourne, AUSTRALIA

## Abstract

Video games are increasingly used in vision science and clinical interventions, particularly in the treatment of amblyopia. Among them, action video games have shown promise in enhancing visual functions such as attention, spatial resolution, and contrast sensitivity. However, the classification of games in current studies typically relies on broad commercial genre labels, which lack functional specificity and fail to capture the perceptual, cognitive, and motor demands relevant to therapeutic use. This imprecision can lead to suboptimal game selection and limit comparability across studies. To address this gap, we developed a data-driven framework to classify commercial video games based on functional load profiles. Twelve experts evaluated seven games across nine dimensions derived from prior literature on action video games, including *Perceptual Load*, motor demands, *Working Memory*, and attentional control. We applied Multidimensional Scaling and K-means clustering to group games based on similarity ratings, and validated the structure using Principal Component Analysis. Three distinct clusters emerged: (1) Action video games with high motor and *Perceptual Load* (e.g., *Call of Duty*, *Unreal Tournament*); (2) puzzle and arcade games with moderate visuomotor and cognitive engagement (e.g., *Tetris*, *Pac-Man*); and (3) low-demand simulation games (*The Sims*). Notably, Tetris reflected moderate visuomotor but higher cognitive demands, confirming its hybrid profile. This multidimensional classification provides a reliable and objective tool to guide therapeutic video game selection and development, offering a valuable alternative to the subjective genre-based selection of video games in both research and clinical applications.

## Introduction

Video games have emerged as promising tools for enhancing cognitive and visual abilities in both basic and applied research contexts. However, not all video game genres share the same properties or cognitive benefits. In particular, Action Video Games (AVGs) have consistently demonstrated improvements in visual attention and related functions [1–6], and several studies have leveraged this potential to enhance visual abilities in amblyopia [7–14]. Traditional methods such as occlusion therapy often face limitations in compliance and do not target binocular integration [15–21]. In contrast, AVG-based interventions, especially those using dichoptic formats, have shown promise in improving not only visual acuity but also stereopsis and other aspects of binocular vision [2,7,11,15,18,22–29]. These approaches capitalize on the engaging nature of interactive digital environments to stimulate visual plasticity, often in shorter timeframes than patching. Thus, video games represent a compelling alternative or complement to traditional amblyopia treatments, especially when selected to match specific cognitive and visual demands.

Important considerations must be addressed when using video games in interventional studies aimed at improving cognitive abilities through behavioral training. First, the characterization of video games is essential, as each demand specific cognitive and motor skills. The Bavelier Lab has used a questionnaire to asses participants’ video game history [1,30]. Participants were classified based on game genre and average weekly playtime during the year preceding the study.

The choice of commercial video games is critical. AVGs are typically fast-paced, first-person shooters (FPS) like *Medal of Honor* or *Call of Duty*, considered “ideal” AVGs. In contrast, non-action video games (NAVGs) like *The Sims* or *Tetris* differ significantly in perceptual, motor, and cognitive demands. Some studies have addressed this gap by defining key characteristics an AVG should include [4,31]: a) fast pace-time events, b) attention distribution across the peripheral visual field, c) shifting focus across location, d) divided attention, and e) avoidance of automatization. Games fulfilling these criteria can be classified as AVGs for interventional purposes.

An important advantage of this classification is its flexibility to include child-friendly AVGs, making interventions accessible to younger participants. However, the classification remains subjective, and the diversity of game genres complicates genre assignment. Classification systems such as the multidimensional scaling (MDS) technique provide a visual representation of pairwise distances between video game elements and can be represented in multiple dimensions [32,33].

In this study, we present a classification using an MDS combined with inter-rater reliability clustering analysis, applied to different video games previously used to improve visual function in amblyopia. This method could assist researchers in identifying the most appropriate games for visual training studies. It also offers a clear reference point to compare the cognitive, perceptual, and motor demands of different games, helping investigators select those that best fit the needs of visual rehabilitation programs.

## Methods

To systematically assess the perceptual and cognitive characteristics of video games, we selected nine dimensions. Eight of these have been commonly cited to define AVGs [4,31], while a ninth, level progression, was introduced by our research team as an additional methodological contribution. This dimension captures the dynamic increase in difficulty across gameplay stages, reflecting not only evolving cognitive demands but also sustained engagement and adaptability. Its inclusion is particularly relevant for assessing a game’s potential to stimulate learning, strategic *Planning*, and motivation, factors of special interest in therapeutic and rehabilitation contexts. The dimensions included: (1) *Scene Rhythm*, (2) *Perceptual Load*, (3) *Motor Load*, (4) *Working Memory*, (5) *Planning*, (6) *Target Accuracy*, (7) *Divided Attention*, (8) *Level of Distraction*, and (9) *Level Progression* (i.e., increasing game difficulty across stages). Each dimension was rated on a 5-point Likert scale, with 1 indicating the lowest and 5 the highest demand. Full descriptions of each dimension are provided in S2 Table in S1 File.

The video games included were *Call of Duty*, *Medal of Honor, Unreal Tournament, Tetris, Pong, Pac-Man, and The Sims*, are commercially available. Most were selected based on prior use in amblyopia research (S3 Table in S1 File), particularly in studies involving monocular or binocular gameplay interventions [2,7,8,10,34–40]. A few titles, though not previously employed in amblyopia research, were included for their comparable characteristics to commercial AVGs.

Two hours of gameplay for each game were sourced from publicly available recordings on YouTube® (see S4 Table in S1 File for video links). These videos were viewed by a panel of expert raters to evaluate each game’s performance across the nine dimensions.

Twelve expert raters independently evaluated the same gameplay videos across all nine dimensions. No consensus discussion was conducted during this stage. These direct ratings formed the basis for all statistical analyses, ensuring broad coverage of perspectives while minimizing group conformity effects.

The panel of twelve expert raters included professionals with advanced academic training (PhD or MD) in visual neuroscience, optometry, or experimental psychology. The experts were recruited through purposive sampling based on their academic background and demonstrated expertise in visual cognition and assessment. All raters had prior familiarity with cognitive-behavioral evaluation tools and action video game paradigms. Before rating, they received detailed written materials outlining the nine dimensions, along with illustrative examples, to ensure a shared interpretative framework. The complete guideline and rating questionnaire provided to experts are available in the S1 Methods in S1 File.

Descriptive statistics were computed for each game and dimension. Cronbach’s alpha was used to assess internal consistency, calculated separately for each expert. Weighted Cohen’s Kappa assessed inter-rater agreement across 66 expert pairs. Global scores for each game were computed by summing ratings across all dimensions and experts. To compare each game’s performance against the average of others, Wilcoxon signed-rank tests for paired samples were conducted. Additionally, Friedman tests were used to examine overall differences between games and across dimensions, followed by Nemenyi post hoc comparisons to identify specific pairwise contrasts.

To explore similarity between games, a dissimilarity matrix was constructed from expert ratings, serving as input for MDS. We also conducted Principal Component Analysis (PCA) to interpret latent structure and identify cognitive and perceptual contributors to variability. MDS enabled visualization of game similarity and unsupervised clustering via K-means, while PCA highlighted underlying structure. All statistical analyses were conducted in R (v2024.12.1 + 563) using RStudio.

### Ethical considerations

This study involved twelve expert raters who evaluated gameplay videos of commercial video games. According to the ethical framework of the Universitat Politècnica de Catalunya (CEUPC) [41] and in line with other institutions in the field, research that does not involve interaction with human participants or the collection of personal or sensitive data does not require formal ethics approval. As the study only involved anonymous expert ratings without personal data collection [42,43], it was therefore exempt from ethics review. All participants provided written informed consent before completing the questionnaire, in accordance with widely accepted research ethics standards.

## Results

### Initial descriptive analysis

Twelve expert raters evaluated seven commercially available video games across nine perceptual and cognitive dimensions. Descriptive statistics revealed a clear distinction between AVGs and NAVGs (Table 1). AVGs (*Call of Duty*, *Medal of Honor, Unreal Tournament*) consistently received high scores in key perceptual and attentional features, including *Scene Rhythm*, *Perceptual Load*, *Motor Load*, and *Divided Attention*, with average ratings around 4.5 (±0.5). These findings suggest high visual-processing demands.

**Table 1:** Mean and standard deviation of expert ratings for each video game across nine dimensions. R, Rhythm; PL, Perceptive Load; ML, *Motor Load*; WM, Working Memory; P, Planification; TA, Target Accuracy; DA, Divided Attention; D, Distraction; Level Progression.

Video Game	R	PL	ML	WM	P	TA	DA	D	LP
**Call of Duty**	4.8 ± 0.2	4.8 ± 0.3	4.7 ± 0.4	2.1 ± 0.5	2.6 ± 0.6	4.8 ± 0.3	4.7 ± 0.3	4.5 ± 0.4	2.3 ± 0.6
**Medal of Honor**	4.6 ± 0.3	4.7 ± 0.2	4.6 ± 0.3	2.4 ± 0.6	2.9 ± 0.5	4.6 ± 0.3	4.5 ± 0.4	4.4 ± 0.3	2.6 ± 0.5
**Unreal Tournament**	4.7 ± 0.3	4.6 ± 0.2	4.7 ± 0.2	2.3 ± 0.6	2.5 ± 0.7	4.8 ± 0.2	4.6 ± 0.3	4.3 ± 0.4	2.4 ± 0.5
**Tetris**	3.0 ± 0.6	3.0 ± 0.5	2.97 ± 0.6	4.0 ± 0.6	4.0 ± 0.6	2.5 ± 0.5	3.0 ± 0.7	3.0 ± 0.6	3.3 ± 0.7
**Pac-Man**	2.5 ± 0.8	2.7 ± 0.6	2.6 ± 0.7	3.5 ± 0.7	3.6 ± 0.8	2.2 ± 0.6	2.8 ± 0.5	2.6 ± 0.5	3.1 ± 0.6
**Pong**	2.3 ± 0.7	2.1 ± 0.7	2.0 ± 0.5	2.8 ± 0.6	2.9 ± 0.5	2.0 ± 0.7	2.5 ± 0.6	2.5 ± 0.5	2.8 ± 0.6
**The Sims**	1.9 ± 0.6	1.8 ± 0.5	1.8 ± 0.5	1.11 ± 0.5	1.11 ± 0.4	1.9 ± 0.6	2.2 ± 0.4	2.3 ± 0.5	1.44 ± 0.4

In contrast, NAVGs (*Tetris, Pac-Man, Pong, and The Sims*) received lower scores in these domains, with average ratings ranging from 2.5 to 3.0 (±0.8), indicating lower perceptual and motor demands. Notably, *Tetris* exhibited higher values in *Planning* and *Working Memory* (mean ≈ 4.0 ± 0.6), suggesting that some NAVGs engage complex cognitive strategies despite their simpler visual dynamics.

### Internal consistency of the ratings (Cronbach’s Alpha)

The reliability of expert evaluations was assessed using Cronbach’s alpha for each of the 12 raters. Results showed high consistency across all dimensions, with alpha values ranging from 0.89 to 0.97, and a global average of 0.93, indicating excellent internal reliability. These findings confirm that experts applied consistent criteria across video games, validating the use of the nine defined dimensions. Dimension-specific alpha values (Table 2) further reinforce the robustness of the evaluation protocol.

**Table 2:** Cronbach’s Alpha for Expert Ratings Across Dimensions.

Dimension	Cronbach’s Alpha Range
**Rhythm**	0.91 - 0.96
**Perceptive Load**	0.92 - 0.97
**Motor Load**	0.90 - 0.95
**Working Memory**	0.88 - 0.95
**Planification**	0.90 - 0.98
**Target Accuracy**	0.92 - 0.97
**Divided Attention**	0.89 - 0.94
**Distraction**	0.89 - 0.94
**Level Progression**	0.90 - 0.94

### Global rating analysis by video game

Overall performance was obtained by summing all expert ratings across dimensions for each video game. The highest scores corresponded to Unreal Tournament, *Call of Duty*, and Medal of Honor, while the lowest were The Sims, Pac-Man, and Pong. Statistically significant differences were observed in all pairwise comparisons, except for *Call of Duty* vs. *Medal of Honor* (p = 0.725) and Tetris vs. Pong (p = 0.142). These differences were assessed using Wilcoxon signed-rank tests for paired samples. Complete descriptive results and pairwise Wilcoxon test comparisons are provided in Supplementary S19 and S20 Tables in S1 File.

### Inter-expert agreement (Weighted Cohen’s Kappa)

To assess inter-rater agreement and minimize subjectivity, Weighted Cohen’s Kappa was calculated across all 66 expert pairings. The results showed that 83.33% of expert pairs achieved a Kappa value of ≥ 0.5, indicating moderate to good agreement. Furthermore, 54.54% achieved ≥ 0.6 (good agreement), and 10.61% ≥ 0.7 (strong agreement). Agreement levels were higher in objective dimensions (e.g., *Scene Rhythm*, *Motor Load* and lower in more interpretative ones (e.g., *Planning*, *Distraction*). Overall, these results support the reliability and validity of the expert-based assessment. The 66 pairings were obtained by calculating all possible unique pairs among the 12 expert raters, ensuring a comprehensive estimate of inter-rater agreement. All individual Kappa values and their corresponding 95% confidence intervals are presented in S4 Table in S1 File.

### Friedman and Nemenyi tests: Between-game and within-game comparisons

To detect domain-specific differences, two complementary Friedman analyses were performed: the first tested for between-game differences within each cognitive-perceptual dimension, while the second examined within-game differences across dimensions. In the between-game approach, Friedman tests were conducted for each dimension. All dimensions showed significant main effects of video game (χ² range = 47.19–65.96, p < 0.001) (S6 Table in S1 File), confirming that games differed significantly in their cognitive-perceptual profiles. Post hoc Nemenyi tests revealed consistent differences, especially between FPS games and games with lower cognitive-perceptual demands. For example, *Call of Duty* and *Unreal Tournament* significantly outperformed *The Sims* across nearly all dimensions (p < 0.001), especially in *Motor Load*, *Scene Rhythm*, and attentional components. Although not classified as an AVG, *Tetris* displayed hybrid profile, significantly differing from *The Sims* in *Planning* and *Level Progression* (S7 Table in S1 File).

In the within-game approach, a separate Friedman test was performed for each video game across all nine dimensions. All games displayed significant internal differences between dimensions (χ² range = 21.16–76.96, p < 0.01) (S8 Table in S1 File), indicating that each game possesses a distinct pattern of cognitive-perceptual demands. Nemenyi post hoc tests (S9 Table in S1 File) identified specific dimension pairs responsible for these differences. In FPS games, *Working Memory* and *Level Progression* were consistently lower than perceptual and attentional demands; in Tetris, *Planning* and perceptive load were higher than *Distractio*n and *Divided Attention*; and for Pong and Pac-Man, Working Memory was notably lower than other domains.

Together, these results highlight the multidimensional and non-uniform functional profiles of commercial video games, both across and within titles. This comprehensive approach demonstrates not only that different games emphasize distinct cognitive and perceptual skills, but also that each game has its own unique distribution of cognitive-perceptual demands.

### Multidimensional scaling (MDS) analysis and clustering

Euclidean distances were computed using the original (non-standardized) expert rating vectors across the nine dimensions, in order to preserve the relative magnitude and structure of the raw perceptual-cognitive scores. To explore the relationships between video games, MDS and K-means clustering analyses were applied to expert ratings. Non-metric MDS was used to reduce data dimensionality and visually represent similarity patterns among video games. Dimension 1 primarily represents visuomotor and *Perceptual Load*, separating fast-paced action games from slower simulation titles, while Dimension 2 reflects cognitive and attentional demands derived from expert ratings. The two and three-dimensional configurations explained 95.49% and 97.94% of the variance, respectively.

Rhythm of scenes, *Perceptual Load*, and *Motor Load* reached the maximum observed values (4.83), suggesting that certain games demand particularly high levels of visuomotor engagement. Conversely, *Working Memory* and *Planning* exhibited lower mean values, indicating relatively reduced demands in these domains across the game set (S10 Table in S1 File).

The optimal number of clusters (n = 3) was determined using the elbow method and validated through silhouette analysis (mean silhouette coefficient = 0.67). The final clustering revealed the following groupings:

- Cluster 1 (n = 3): Games with high motor and *Perceptual Load*.
- Cluster 2 (n = 3): Games with moderate cognitive and perceptual demand. Within this cluster, Tetris exhibits a hybrid profile, combining moderate visuomotor with higher cognitive and *Planning* load
- Cluster 3 (n = 1): Games with low perceptual and *Motor Load*, characterized by minimal demands on speed and accuracy.

A Euclidean distance matrix (S11 Table in S1 File) confirmed inter-game similarity patterns. Matrix values reflected the proximity between each video game pair within the MDS-generated multidimensional space. Cluster-level analysis revealed that Cluster 3 was the most distinct (mean distance = 2.03), while Clusters 1 and 2 exhibited higher proximity (1.12). Within-cluster distances further supported the coherence of Cluster 2 (mean = 0.912) and the singular profile of Cluster 3 (mean = 1.796).

**Fig 1:**
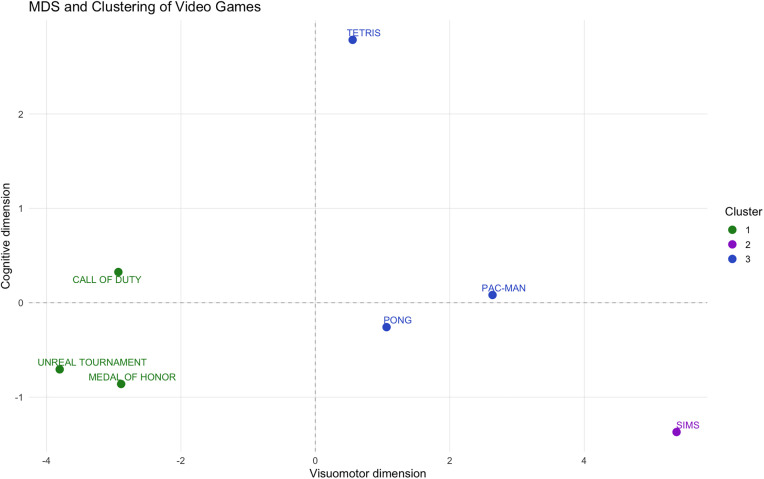
Two-dimensional MDS plot of video games based on expert ratings. Colors indicate K-means clusters (n = 3). The model captures 95.49% of the variance (Dim1: 83.77%, Dim2: 11.72%; Stress ≈ 0). The MDS coordinates are listed in Supplementary S16 Table in S1 File, and the corresponding Euclidean distance matrix in S18 Table in S1 File.

**Fig 2:**
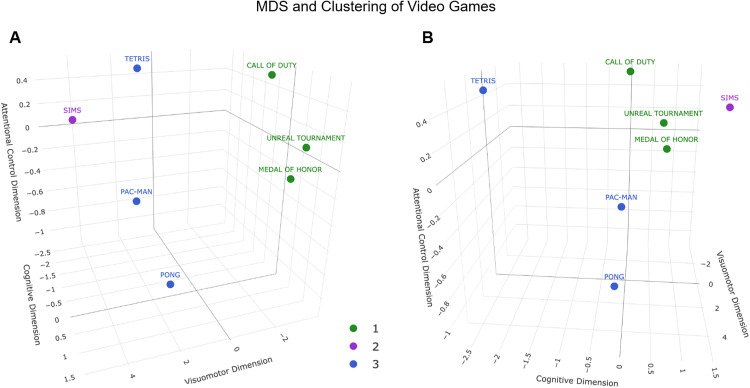
Three-dimensional MDS representation of video games with K-means clustering. (A) Frontal view of the 3D configuration in the plane defined by Dim1 (visuomotor load) and 2 (cognitive demand). B) Rotated view of the same configuration, highlighting the contribution of Dimension 3 (attentional control). Colors indicate K-means clusters (n = 3). The model explains 97.94% of the variance (Dim1: 83.77%, Dim2: 11.72%, Dim3: 2.45%; Stress ≈ 0). Dim1 represents visuomotor load, Dim2 represents cognitive demand, and Dim3 represents attentional control. The MDS coordinates are listed in Supplementary S17 Table in S1 File, and the corresponding Euclidean distance matrix in S18 Table in S1 File.

Figs 1 and 2 illustrate the spatial distribution of the video games as revealed by MDS analysis. In the 2D plot, FPS games cluster within Cluster 1, reflecting their high perceptual and motor demands. Tetris appears in a distinct position in the 2D MDS space, reflecting moderate visuomotor demands and relatively higher cognitive load compared with other titles. This places it between the high-load FPS cluster and the low-demand games. In contrast, Pac-Man and Pong are located further apart, consistent with their lower visuomotor complexity. The 3D representation offers clearer group separation, with FPS games remaining tightly clustered, and *Tetris* shifting toward a unique position closer to strategy-oriented games. These findings suggest a nuanced relationship between game categories and their cognitive-perceptual profiles, supporting the need for more refined classification in experimental research.

In the 3D configuration, the third dimension (Dim3) represents attentional control, derived primarily from the *Distraction* variable, and captures variance related to interference resistance and sustained focus. This component complements the visuomotor (Dim1) and cognitive (Dim2) dimensions, providing a more complete representation of the attentional–perceptual space.

### Validation of dimension structure and cluster robustness

**Fig 3:**
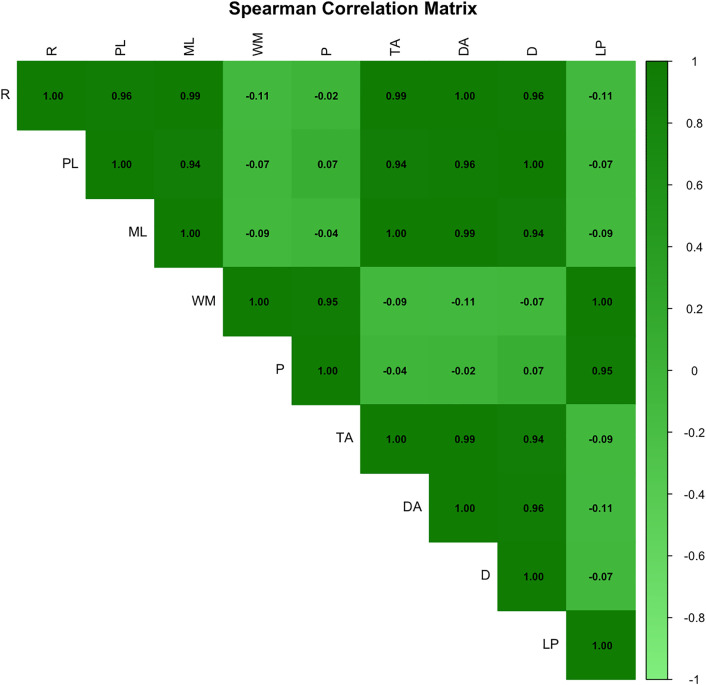
Spearman correlation matrix across the nine dimensions. Darker shades represent stronger positive correlations.

A Spearman correlation matrix was computed to assess relationships between dimensions (Fig 3). High positive correlation values were observed between *Scene Rhythm* and *Perceptual Load* (ρ = 0.96), *Scene Rhythm* and *Motor Load* (ρ = 0.96), and *Perceptual Load* and *Motor Load* (ρ = 0.93), suggesting potential overlap in visuomotor demands. The *Level Progression* dimension showed generally low correlations with other variables, the highest being with *Working Memory* (ρ = 0.32) and *Planification* (ρ = 0.22). Correlations with *Rhythm* (ρ = 0.13), *Perceptual Load* (ρ = 0.19), *Motor Load* (ρ = 0.09), and *Distraction* (ρ = 0.07) were weak or negligible, suggesting it captures a distinct cognitive aspect.

A hierarchical clustering using Ward’s method (S12 Fig in S1 File) reinforced the K-means results [44,45], consistently grouping AVGs together and separating lower-demand games like The Sims and Pac-Man. Silhouette analysis supported cluster cohesion (average silhouette width = 0.46), with Cluster 2 exhibiting strong internal consistency (0.61) and Cluster 3 showing a silhouette width of 0.00, consistent with its unique profile.

These results reinforce the validity of the MDS and K-means classifications, supporting the existence of distinct functional categories among the video games based on their cognitive and perceptual profiles.

### Principal Component Analysis (PCA) of expert ratings across cognitive dimensions

To complement the MDS, PCA was performed to extract latent structures underlying the ratings. Principal Component 1 (PC1) (Dimension 1) explained 68.18% of the variance and was primarily defined by *Scene Rhythm*, *Perceptual Load*, *Motor Load*, *Divided Attention*, and *Target Accuracy*. Principal Component 2 (PC2) (Dimension 2) explained an additional 14.31% and reflected level progression, *Working Memory*, and *Planning* (S13 Fig in S1 File).

**Fig 4:**
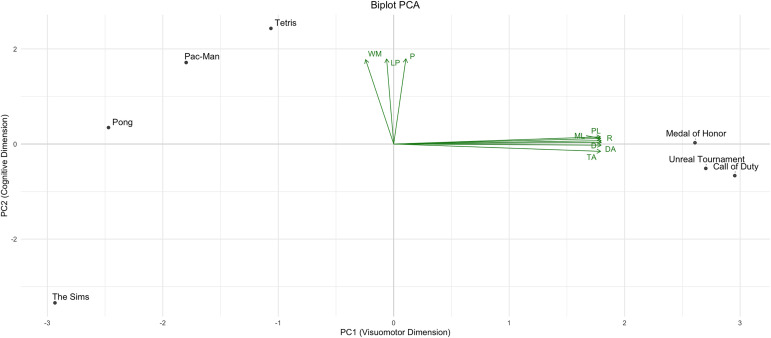
PCA biplot of expert ratings across nine cognitive and perceptual dimensions. Points represent individual video games, and green arrows indicate the direction and relative contribution of each variable to the PCA space. PC1 (68.18% variance explained) is shown on the x-axis, and PC2 (14.31% variance explained) on the y-axis. R, Rhythm; PL, Perceptive Load; ML, Motor Load; WM, Working Memory; P, Planification; TA, Target Accuracy; DA, Divided Attention; D, Distraction; LP, Level Progression.

The PCA biplot (Fig 4) revealed clear spatial separation: FPS games clustered in the lower-right quadrant, reflecting high visuomotor demands. *Tetris* occupied a central-upper position, indicating higher-order cognitive engagement. *Pac-Man* and *Pong* appeared in the upper-left, and *The Sims* in the lower-left quadrant, reflecting minimal cognitive and perceptual demands.

These results align closely with those from MDS and K-means clustering, offering convergent evidence for a multidimensional classification framework. Complete statistical details are provided in S14 and S15 Tables in S1 File.

## Discussion

There is a growing body of evidence that AVGs can enhance various visual skills in individuals with normal vision, including spatial and temporal resolution, contrast sensitivity, and selective attention (for reviews, see Bediou et al.,2018; Green & Bavelier, 2003; Achtman et al., 2008; Bavelier et al., 2012) [3–5,46]. These findings have prompted their application in amblyopia rehabilitation, as such video games require rapid visual processing, precise motor control, and high attentional load, key aspects in visual recovery [2,47]. While several video games have been employed in previous studies, selection has often relied on subjective criteria, such as commercial genre or game availability, without a systematic assessment of the perceptual, cognitive, or motor demands involved in each game. Although AVGs have demonstrated the potential to improve both visual and cognitive functions, the absence of a functional, data-driven classification system may limit their therapeutic impact and hinder comparability across studies.

### Constructing and interpreting the functional space of video games

To overcome the limitations inherent in subjective video games classification systems, this study introduces an objective framework grounded in the functional characteristics of games, with particular emphasis on their perceptual, cognitive, and motor demands. By combining MDS and K-means clustering, we grouped commercially available video games across nine expert-rated dimensions. Eight were drawn from the existing literature (4), and one, Level progression, was introduced as a novel methodological contribution.

This data-driven approach offers a robust alternative to conventional genre-based classifications, enabling assessments rooted in actual functional load. Our analysis confirmed the effectiveness of combining MDS and K-means clustering in organizing games into a coherent and interpretable space. This structure facilitates targeted therapeutic selection, aligning game profiles with individual clinical needs. A key methodological contribution is the demonstration of how subjective similarity ratings between video games can be transformed into continuous representations using MDS.

Each dimension reflects fundamental player and game interactions and helps define a functional profile for each title. Level progression, introduced here, captures the gradual increase in difficulty as the player advances through the game. Although not included in existing classification frameworks, this feature is considered crucial to learning processes and skill acquisition. Evidence shows that progressive difficulty enhances cognitive engagement, facilitates flow states, and improves both skill retention and transfer [48–51]. In addition, adaptive challenge levels have been shown to increase neural activation in brain regions involved in executive function and motor control, thereby amplifying training effects and promoting generalization to novel contexts [52]. This added dimension captures a key aspect of a game’s structural complexity beyond immediate perceptual or *Motor Load*. Its inclusion addresses a therapeutic need: selecting games that not only engage users, but also support progressive functional development.

The correlation analysis (Fig 3) further clarifies the functional meaning of the three dimensions identified in the MDS structure. *Rhythm*, *Perceptual Load*, and *Motor Load* were highly correlated (ρ > 0.9), defining the visuomotor axis (Dim1). *Working Memory*, *Planning*, and *Level Progression* were moderately correlated (ρ ≈ 0.3), contributing primarily to the cognitive or *Planning* axis (Dim2). In contrast, *Distraction* exhibited very low correlations with all other variables (ρ < 0.1), indicating that it explained an independent portion of the variance, largely accounting for the third dimension (Dim3). This axis, which we reinterpret as attentional control, may capture a combination of attentional stability, interference resistance, and sustained focus. Alternatively, it might also encompass motivational or environmental factors, such as engagement level or visual clutter, which influence attentional load independently of visuomotor or cognitive demands. Overall, this dimension provides a complementary attentional component to the visuomotor and cognitive dimensions, offering a more complete representation of the functional space of video games.

As evidenced by the correlation analysis (Fig 3), *Level Progression* appears to be a relatively independent dimension within the cognitive-perceptual space. In contrast to *Scene Rhythm*, *Perceptual Load*, and *Motor Load*, which were highly intercorrelated and likely reflect shared visuomotor demands, *Level Progression* showed only modest associations with *Working Memory* (ρ = 0.32) and *Planification* (ρ = 0.22). These findings suggest that this dimension may be more closely related to higher-order cognitive processes, such as strategic *Planning* and temporal integration, rather than immediate perceptual or motor requirements.

Our MDS revealed cohesive clustering among AVGs, while strategy and puzzle games showed greater dispersion in their cognitive characteristics. The resulting dimensional space, validated via PCA, exhibited two main trends. The first reflects cognitive load and strategic *Planning*, distinguishing games that require high levels of *Working Memory* and structured decision-making. The second primarily reflects motor and attentional demands, grouping games that require rapid responses, high precision, and sustained *Divided Attention*. For instance, *Call of Duty* and *Unreal Tournament* occupy the high end of the motor-attentional axis, whereas *Tetris* stands out for its cognitive profile, emphasizing *Planning* and *Working Memory*.

These findings confirm that the functional space derived from MDS effectively captures the perceptual and cognitive complexity of video games, enabling precise classification for therapeutic applications. Games with high motor-attentional load may enhance visuomotor coordination and attentional flexibility, while cognitively demanding games support *Planning* and memory. This approach transcends commercial labels, offering a more personalized and functionally grounded selection framework for rehabilitation contexts.

### Functional clustering of video games and therapeutic implications

The first cluster, identified through K-means, included video games with high perceptual and motor demands, primarily FPS such as *Call of Duty*, *Medal of Honor*, and *Unreal Tournament*. These games are characterized by fast-paced interactions, continuous visual stimulation, precise motor responses, and sustained *Divided Attention* across multiple dynamic elements. This functional profile reflects intense activation of visuomotor coordination, rapid target detection, and motor accuracy, positioning these games as ideal candidates for visual rehabilitation.

Several studies have demonstrated that FPS games can promote visual plasticity in amblyopia. For instance, (2011) reported improvements in visual acuity (~0.3 logMAR) and stereoacuity (53.6%) after 40–80 hours of gameplay with *Medal of Honor* [11]. Similar gains were observed with *Unreal Tournament* in both monocular [13] and dichoptic formats [7]. Collectively, these findings suggest that the games in Cluster 1 not only share high functional demands but also demonstrate significant potential to promote visual plasticity.

While grouped together functionally, PCA analysis revealed subtle intra-cluster differences. As shown in Fig 4, the three FPS titles cluster at the far right of PC1, indicating strong associations with motor and perceptual dimensions. However, *Unreal Tournament* appears slightly further out in the PCA biplot, suggesting it may impose even higher demands for rhythm and precision compared to the other titles. This internal variability highlights that even within a shared functional cluster, games can differ significantly in how they engage specific perceptual and motor functions. Consequently, although the conventional AVG/NAVG classification used in prior studies provides a useful starting point, a more nuanced, data-driven selection process may be necessary to optimize therapeutic outcomes based on each game’s specific functional profile.

The second cluster included games primarily characterized by cognitive demands and moderate perceptual and motor requirements, such as *Tetris*, *Pac-Man*, and *Pong*, which share core features such as the need to anticipate movement, make rapid decisions, and manipulate visual stimuli using controlled input, though without the intense sensory stimulation typical of FPS games.

This cluster also exhibited the greatest functional dispersion, as shown in the PCA biplot (Fig 4), indicating substantial heterogeneity in the dimensions activated by each game. For example, *Tetris* appears at the upper end of PC2, associated with *Planning*, *Working Memory*, and *Level Progression*, while *Pac-Man* and *Pong* are located closer to the center of the plot, reflecting a simpler, more reactive cognitive profile.

The position of *Tetris* within the MDS space is particularly revealing. Although it does not cluster with the FPS games, its proximity to them on specific dimensions suggests that it shares key attentional and visuomotor properties. *Tetris* is defined by the need for active visual monitoring, spatial precision, and sustained movement coordination under time pressure. In contrast to FPS games, which rely on *Divided Attention* within dynamic 3D environments, *Tetris* requires focused spatial reasoning and precise execution on a structured 2D grid, engaging attentional systems in a functionally similar, though structurally distinct, manner. This hybrid profile may explain why *Tetris* activates mechanisms similar to those activated in AVGS, despite its apparent simplicity.

A dichoptic version of *Tetris* has also shown efficacy, with improvements in acuity (~0.07–0.21 logMAR) and stereoacuity in children and adults [35]. These results suggest that *Tetris* cannot reliably be considered a neutral placebo, particularly when delivered in binocular or dichoptic formats. Accordingly, the video games in Cluster 2 occupy an intermediate functional position between high-demand AVGS and low-stimulation control games. Their use in therapeutic interventions or experimental paradigms should be based on a nuanced understanding of their cognitive load and perceptual structure. Despite their apparent simplicity, these games may engage visual and executive processes that are highly relevant to amblyopia treatment.

Nevertheless, despite similarities in attentional engagement, structural differences between *Tetris* and FPS games matter. FPS games involve immersive, high-speed 3D coordination, while *Tetris* involves slower, focused manipulation in 2D. These distinctions may affect therapeutic outcomes, underscoring the need for structural as well as functional matching. The third cluster identified through K-means consisted of a single game, *The Sims*, which showed globally low perceptual, motor, and cognitive demands. It involves slow-paced, non-reactive interactions with minimal strategic or memory requirements. In the MDS and PCA space, it appeared as an outlier, validating its minimal engagement.

Clinically, *The Sims* may be a suitable candidate for passive control or placebo conditions. Notably, *The Sims* was used as a control game in the study by Li et al. (2011), where it produced a 1.5 line improvement in visual acuity in a very small sample (n = 10) [11]. Although often treated as inert, low-demand games may influence results via motivation, engagement, or compliance. Therefore, their use as controls requires justification based on functional criteria, not convenience, to isolate active effects in experimental designs.

### Functional heterogeneity within individual games

Beyond inter-game differences, our within-game analyses revealed that each video game also displays internally heterogeneous cognitive-perceptual profiles, as confirmed by the Friedman tests (S7 Table in S1 File). These results indicate that even within a single title, cognitive demands are unevenly distributed across dimensions. For instance, FPS games such as Unreal Tournament and Medal of Honor showed significantly lower scores in *Working Memory* and *Level Progression* compared to perceptual and attentional components. Tetris, often considered cognitively homogeneous, exhibited higher demands in *Planning* and *Perceptual Load* relative to *Distraction* and *Divided Attention*. Even games with simpler structures, like Pong and Pac-Man, demonstrated selective weaknesses, particularly in *Working Memory*. These intra-game contrasts, supported by Nemenyi post hoc tests (S8 Table in S1 File), highlight that functional diversity exists not only between games but also within them, suggesting that therapeutic game selection should consider a game’s internal cognitive structure, not just its overall classification. This nuance reinforces the value of a multidimensional evaluation framework over simplistic genre-based categorizations.

### Convergent validity and structural robustness

The classification framework showed high internal consistency and inter-rater reliability, underscoring its methodological strength. Expert ratings yielded Cronbach’s alpha values from 0.89 to 0.97 (mean α = 0.93), confirming that the nine dimensions captured coherent and functionally relevant features. Weighted Cohen’s Kappa further supported reliability: 83.33% of expert pairs had values ≥ 0.5, and 54.54% exceeded 0.6, especially on more objective dimensions like *Scene Rhythm* and *Motor Load*.

Structural validation was achieved through multiple converging methods. The 2D and 3D MDS solution explained 95.49% and 97.94% of the variance (Stress ≈ 0), and K-means clustering produced a mean silhouette score of 0.67, indicating well-separated groups. Hierarchical clustering using Ward’s method [44,45], supported this organization, showing strong cohesion for Cluster 2 (silhouette = 0.73) and distinct separation for Cluster 3 (silhouette = 0.00).

Together, these findings confirm that MDS and K-means clustering provide a reliable and interpretable structure for classifying video games based on functional load. Unlike commercial genres, this empirical framework captures underlying perceptual, cognitive, and motor characteristics, offering a more precise tool for therapeutic selection or experimental design.

### Limitations

Despite the methodological rigor, this study has several limitations. First, the number of video games was small (n = 7), and all titles were commercially available games selected based on their prior use in amblyopia research. This may limit representativeness, especially of therapeutic games or underexplored genres. Only PC and console games were included, excluding emerging platforms like virtual reality (VR) games, which are gaining traction in clinical contexts. Also, Cluster 3 included only a single video game (The Sims), limiting its statistical robustness. Furthermore, ratings were based on pre-recorded gameplay videos, not interactive play, which might have restricted detection of features like adaptive difficulty or dynamic user feedback.

Finally, while the number of expert raters (n = 12) is consistent with accepted standards in expert judgment research, it may nonetheless limit statistical power to detect small differences between games, especially in more interpretative dimensions, thus increasing the potential for type II errors. However, panels of 10–15 experts are methodologically supported in the literature as sufficient for ensuring validity and reliability when combined with Likert-type scales and standard validation metrics such as Cronbach’s alpha and Cohen’s Kappa [53]. In our study, expert selection was rigorous, preceded by a calibration phase, and supported by internal consistency and agreement metrics to enhance reliability.

### Clinical applications and future research directions

Classifying video games based on their perceptual, cognitive, and motor demands offers a structured and clinically relevant framework to inform therapeutic interventions in amblyopia. By aligning game characteristics with specific visual and cognitive rehabilitation objectives, this approach enables the design of more targeted and engaging treatments, particularly in pediatric settings where adherence and motivation are critical.

From a research perspective, the proposed framework allows for standardized comparisons across studies, facilitating cross-study synthesis and supporting inclusion in systematic reviews and meta-analyses.

Future research should expand the pool of analyzed games to include a broader diversity of games, platforms, and purpose-designed therapeutic games, thereby enhancing ecological validity. Additionally, examining the relationship between individual game dimensions, such as *Motor Load*, *Divided Attention*, or strategic *Planning*, and specific clinical outcomes (e.g., visual acuity, stereoacuity, contrast sensitivity) will help refine intervention strategies. The influence of moderating variables, including age, baseline visual abilities, and duration of exposure, should also be investigated. Finally, the dimensional structure proposed here may guide the design of novel therapeutic games tailored to individual needs, promoting a more systematic and evidence-based approach to video game–based rehabilitation.

## Conclusions

This study applied Multidimensional Scaling (MDS) and K-means clustering to establish a functional classification of commercial video games based on their perceptual, cognitive, and motor demands. The resulting framework identified three distinct functional groups: games with high perceptual and *Motor Load*, games with intermediate cognitive demands, and a low-demand group. Our findings show that action video games form a coherent and highly stimulating cluster. In contrast, although Tetris is typically classified as a non-action video game, it exhibited a notably high cognitive load. We propose that future studies using video games to enhance visual function in amblyopia and other areas of visual attention should apply the proposed classification to improve research applications.

## Supporting information

S1 File Supporting Information (S1–S20). (PDF)
