# Supplementary material for: Classification of videogames for amblyopia treatment in perceptive and cognitive domains
Source: PLoS One. 2025 Oct 28;20(10):e0335510. doi: 10.1371/journal.pone.0335510 (PMC12561906; doi:10.1371/journal.pone.0335510)
Supplement: S1 File — (PDF) [file pone.0335510.s001.pdf]

Supplementary Material

S1 Expert Rating Methodology and Questionnaire

S1.1 Expert Rating Procedure

To systematically evaluate the perceptual, cognitive, and motor characteristics of the selected video games, we conducted an expert-based rating procedure using a structured questionnaire and standardized visual materials.

Twelve experts participated in this process, all with advanced training in visual neuroscience, optometry, or experimental psychology. Experts were recruited through purposive sampling based on their academic background and demonstrated expertise in visual cognition and assessment. Each expert independently evaluated the same set of seven commercially available video games used in previous amblyopia research (*Call of Duty, Medal of Honor, Unreal Tournament, Tetris, Pong, Pac-Man,* and *The Sims*).

Each expert received access to 2-hour gameplay videos for each game (see Supplementary Table S3 for video links). The videos were viewed individually, and experts were instructed to evaluate the games according to nine predefined dimensions.

Instructions to Experts

Experts were given the following written instructions before beginning the evaluation:

1. Watch the gameplay videos in the order provided. Observe multiple moments within each recording, as perceptual and cognitive load may vary during gameplay.

2. Rate each game independently across all nine dimensions using a 1–5 Likert scale, where:
   - 1 = Very Low Demand
   - 2 = Low Demand
   - 3 = Moderate Demand
   - 4 = High Demand
   - 5 = Very High Demand
3. Base your ratings solely on the observed game dynamics, not on personal familiarity or subjective preference.
4. Avoid discussing your evaluations with other experts to maintain rating independence.
5. Provide brief optional comments if clarification or justification of a specific rating is required.
6. Once all videos have been evaluated, submit the completed ratings through the provided online form.

Evaluation Framework

Each game was rated according to the nine predefined dimensions:

1. Scene rhythm
2. Perceptual load
3. Motor load
4. Working memory
5. Planning

6. Target accuracy
7. Divided attention
8. Level of distraction
9. Level progression

The operational definitions for these dimensions are detailed in **S1 Table.**

S1.1. Questionnaire

For each video game, experts completed the following table:

Dimension	Rating (1–5)	Comments (optional)
**Scene Rhythm**	☐1 ☐2 ☐3 ☐4 ☐5	
**Perceptual Load**	☐1 ☐2 ☐3 ☐4 ☐5	
**Motor Load**	☐1 ☐2 ☐3 ☐4 ☐5	
**Working Memory**	☐1 ☐2 ☐3 ☐4 ☐5	
**Planning**	☐1 ☐2 ☐3 ☐4 ☐5	
**Target Accuracy**	☐1 ☐2 ☐3 ☐4 ☐5	
**Divided Attention**	☐1 ☐2 ☐3 ☐4 ☐5	
**Level of Distraction**	☐1 ☐2 ☐3 ☐4 ☐5	
**Level Progression**	☐1 ☐2 ☐3 ☐4 ☐5	

S2 Table. Description of the nine sub-categories used for video game classification.

**Rhythm of scenes**	refers to how fast the character needs to make the necessary moves, to avoid obstacles or enemies, or to collect objects with high frequency. In short, the simple response time is evaluated, that is, the time that passes between the perception of the stimulus and the response that is emitted.
**Perceptual load**	it involves precision in collecting objects or enemies to be considered. Included in this section, color aspects, contrast, measurement, speed, unpredictable trajectory and partial occlusion of objects, enemies or environment.
**Motor load**	includes the precision of the fingers and the control with high frequency to execute the movements. It is tied to the rhythm of scenes and therefore also evaluates the simple response time.
**Working memory**	takes into account the need to memorize the previous screens to have more skills or objects on the following screens. This time this memorization typically occurs over a short period (≤2 minutes)
**Planification**	refers to the need for a motor organization and not an automated one to be able to play, that is, a method and structure to follow in order to advance.

**Target accuracy**	refers to the need to take into account specific objectives, such as: objects to collect or avoid enemies, in order to meet the objectives set by the game.
**Divided attention**	includes attention between many things at the same time as it is played. In many cases you have to look at different objectives and also be prepared for others that appear in the next few minutes. It also evaluates the amount of stimuli to watch for at the same time.
**Level of distraction**	includes elements that distract and hinder discrimination, whether objects to collect or enemies to dodge. It is related with the perceptual load and therefore is considered color, contrast, measurement, speed, unpredictable trajectory, the partial occlusion, the character, the climate and the music, among others.
**Level progression**	refers to the gradual increase in difficulty and complexity of the game’s challenges. It includes the introduction of new obstacles, faster rhythms, higher motor and cognitive demands, and more complex objectives.

S3 Table. Representative video games used in previous amblyopia treatment studies, including mode of presentation (monocular, binocular, or dichoptic), study population, and reported visual outcomes. This table served as a reference framework for the selection of the video games analyzed in the present study. VA = *Visual Acuity*; ST = *Stereoacuity*

Author (Year)	Video Game	Mode of Play	Total Hours	VA	ST
**Li et al. (2011)**	Medal of Honor	Monocular	40–80	+1.6 lines (0.3 logMAR)	+53.6%
**Li et al. (2014)**	Tetris, Balloon, Pong, Labyrinth	Dichoptic vs. Sham	4 hr/week for 4 weeks	0.47 ± 0.19 logMAR	0.39 ± 0.03 logMAR
**Hess et al. (2014)**	Tetris	Dichoptic	10	+0.11 logMAR	Not reported
**Vedamurthy et al. (2015)**	Unreal Tournament	Dichoptic	20	+0.14 logMAR	34%
**Birch et al. (2015)**	Tetris	iPad Dichoptic	4 hr/week for 4 weeks	0.43 ± 0.03 logMAR	0.34 ± 0.03 logMAR
**Kelly et al. (2016)**	Adventure Game (iPad)	Binocular (contrast-balanced)	10–18	+0.15 logMAR (2 weeks)	No effect
**Vedamurthy et al. (2016)**	Bug Squashing VR	VR Dichoptic	~35 sessions	Trend (non-significant)	Significant (Randot, PDT)
**Holmes et al. (2016)**	Falling Blocks (iPad)	Binocular	16 weeks	+1.05 lines (binocular)	No significant change

**Rajavi et al. (2016)**	Pac-Man, Snake, Tetris	Binocular vs. Patching	20 min/session, 5x/week	0.34 ± 0.14 logMAR	0.17 ± 0.14 logMAR
**Webber et al. (2016)**	Tetris	Dichoptic	5 hr/week for 5 weeks	0.44 (0.17) logMAR	0.37 (0.16) logMAR
**Žiak et al. (2017)**	Diplopia Game (VR)	Dichoptic (VR)	40	+0.34 logMAR	70% improved
**Gambacorta et al. (2018)**	Unreal Tournament	Monocular/ Dichoptic	20	+0.14–0.18 logMAR	Improved CS
**Kelly et al. (2018)**	Dig Rush (iPad)	Binocular	10	+0.14 logMAR	Minimal or none
**Gao et al. (2018)**	Falling Blocks	Dichoptic vs Placebo	42 (6 weeks)	+0.06 logMAR (active)	No significant difference
**Manh et al. (2018)**	Tetris	Dichoptic	~20	+0.07 logMAR	NS
**Holmes et al. (2019)**	Falling Blocks (iPad)	Binocular	24 weeks	+1.3 lines	No significant change
**Godinez et al. (2021)**	Contrast-balanced games	Binocular	20	Not reported	No change
**Pang et al. (2021)**	Tetris	Dichoptic	24	+0.09 logMAR	+0.40 log arcsec
**Roy et al. (2022)**	Tetris	Dichoptic	20	+0.21 logMAR	+0.10 log arcsec

S4 Table. Links to gameplay videos used for expert evaluation.

Videogame	Youtube® Videos
Medal of Honor®	https://www.youtube.com/watch?v=3JRSNHDy4Vw
Call of Duty®	https://www.youtube.com/watch?v=KQQ9bYrUyPg
Unreal Tournament®	https://www.youtube.com/watch?v=H3kjxp1qDjI_
Tetris®	https://www.youtube.com/watch?v=VNbo1AGqKrI&t=1496s<br>https://www.youtube.com/watch?v=DwZBJhbiz78
Pong®	https://www.youtube.com/watch?v=D96oI9pAHh4
PacMan®	https://www.youtube.com/watch?v=AuoH0vz3Mqk
The Sims®	https://www.youtube.com/watch?v=qqJ6eO51n_o<br>https://www.youtube.com/watch?v=JIqsAqyudoI<br>https://www.youtube.com/watch?v=U7yqhrEPsz8<br>https://www.youtube.com/watch?v=3oKHMj3yUyE<br>https://www.youtube.com/watch?v=1KKIUrYUayg<br>https://www.youtube.com/watch?v=1km601uKqN0<br>https://www.youtube.com/watch?v=cDuqv9NS40s

https://www.youtube.com/watch?v=MOz-kpoVHvo

https://www.youtube.com/watch?v=Ihl7l0BqQLk

S5 Table. Weighted Cohen's Kappa values and 95% confidence intervals for all pairwise combinations of the 12 expert raters (66 unique pairs).

Expert Pair	Weighted Kappa	95% CI Lower	95% CI Upper
**Expert 1 vs 2**	0.92	0.86	0.97
**Expert 1 vs 3**	0.68	0.58	0.79
**Expert 1 vs 4**	0.63	0.52	0.74
**Expert 1 vs 5**	0.65	0.54	0.77
**Expert 1 vs 6**	0.50	0.37	0.63
**Expert 1 vs 7**	0.64	0.54	0.73
**Expert 1 vs 8**	0.79	0.71	0.87
**Expert 1 vs 9**	0.49	0.36	0.61
**Expert 1 vs 10**	0.57	0.45	0.69
**Expert 1 vs 11**	0.67	0.58	0.77
**Expert 1 vs 12**	0.43	0.31	0.56
**Expert 2 vs 3**	0.61	0.50	0.73
**Expert 2 vs 4**	0.66	0.55	0.77
**Expert 2 vs 5**	0.65	0.52	0.77
**Expert 2 vs 6**	0.47	0.33	0.60
**Expert 2 vs 7**	0.63	0.53	0.73
**Expert 2 vs 8**	0.78	0.70	0.87
**Expert 2 vs 9**	0.78	0.70	0.87
**Expert 2 vs 10**	0.58	0.46	0.70
**Expert 2 vs 11**	0.68	0.58	0.78
**Expert 2 vs 12**	0.44	0.32	0.57

**Expert 3 vs 4**	0.66	0.55	0.77
**Expert 3 vs 5**	0.61	0.50	0.72
**Expert 3 vs 6**	0.45	0.32	0.57
**Expert 3 vs 7**	0.60	0.49	0.72
**Expert 3 vs 8**	0.73	0.63	0.84
**Expert 3 vs 9**	0.55	0.43	0.67
**Expert 3 vs 10**	0.64	0.52	0.75
**Expert 3 vs 11**	0.58	0.47	0.70
**Expert 3 vs 12**	0.46	0.33	0.59
**Expert 4 vs 5**	0.59	0.48	0.71
**Expert 4 vs 6**	0.50	0.36	0.65
**Expert 4 vs 7**	0.60	0.50	0.70
**Expert 4 vs 8**	0.76	0.66	0.86
**Expert 4 vs 9**	0.60	0.48	0.72
**Expert 4 vs 10**	0.63	0.51	0.75
**Expert 4 vs 11**	0.67	0.57	0.78
**Expert 4 vs 12**	0.64	0.51	0.77
**Expert 5 vs 6**	0.58	0.46	0.70
**Expert 5 vs 7**	0.53	0.41	0.64
**Expert 5 vs 8**	0.66	0.55	0.77
**Expert 5 vs 9**	0.66	0.55	0.76
**Expert 5 vs 10**	0.69	0.57	0.81
**Expert 5 vs 11**	0.59	0.46	0.71
**Expert 5 vs 12**	0.48	0.35	0.61

**Expert 6 vs 7**	0.40	0.27	0.53
**Expert 6 vs 8**	0.52	0.38	0.66
**Expert 6 vs 9**	0.61	0.49	0.74
**Expert 6 vs 10**	0.54	0.40	0.68
**Expert 6 vs 11**	0.46	0.31	0.60
**Expert 6 vs 12**	0.43	0.28	0.58
**Expert 7 vs 8**	0.68	0.60	0.77
**Expert 7 vs 9**	0.49	0.36	0.62
**Expert 7 vs 10**	0.57	0.45	0.68
**Expert 7 vs 11**	0.64	0.54	0.73
**Expert 7 vs 12**	0.50	0.38	0.62
**Expert 8 vs 9**	0.54	0.41	0.67
**Expert 8 vs 10**	0.62	0.51	0.74
**Expert 8 vs 11**	0.70	0.61	0.80
**Expert 8 vs 12**	0.58	0.45	0.71
**Expert 9 vs 10**	0.61	0.48	0.75
**Expert 9 vs 11**	0.52	0.39	0.64
**Expert 9 vs 12**	0.50	0.38	0.63
**Expert 10 vs 11**	0.65	0.54	0.76
**Expert 10 vs 12**	0.65	0.53	0.76
**Expert 11 vs 12**	0.52	0.39	0.64

S6 Table. Friedman Test Results by Dimension

Dimension	Chi-squared	p-value
**Scene Rhythm**	62.292	< 0.001
**Perceptual Load**	65.375	< 0.001
**Motor Load**	58.978	< 0.001
**Working Memory**	47.189	< 0.001
**Planification**	53.876	< 0.001
**Target Accuracy**	65.960	< 0.001
**Divided Attention**	59.384	< 0.001
**Distraction Level**	64.993	< 0.001
**Level Progression**	57.116	< 0.001

All dimensions showed statistically significant differences between video games ($p < 0.001$). Significant pairwise comparisons from the Nemenyi post hoc tests are detailed in Table S3b.

S7 Table. Significant Nemenyi Pairwise Comparisons by Dimension

Dimension	Game 1	Game 2	p-value
**Scene Rhythm**	PAC-MAN	CALL OF DUTY	0.0348
**Scene Rhythm**	SIMS	CALL OF DUTY	<0.001
**Scene Rhythm**	PAC-MAN	MEDAL OF HONOR	0.0223
**Scene Rhythm**	SIMS	MEDAL OF HONOR	<0.001
**Scene Rhythm**	UNREAL TOURNAMENT	PAC-MAN	0.0006
**Scene Rhythm**	UNREAL TOURNAMENT	PONG	0.0086
**Scene Rhythm**	TETRIS	SIMS	0.0348
**Scene Rhythm**	UNREAL TOURNAMENT	SIMS	<0.001
**Perceptual Load**	PAC-MAN	CALL OF DUTY	0.0001
**Perceptual Load**	PONG	CALL OF DUTY	0.0348
**Perceptual Load**	SIMS	CALL OF DUTY	<0.001
**Perceptual Load**	PAC-MAN	MEDAL OF HONOR	0.0001
**Perceptual Load**	PONG	MEDAL OF HONOR	0.0401

**Perceptual Load**	SIMS	MEDAL OF HONOR	<0.001
**Perceptual Load**	UNREAL TOURNAMENT	PAC-MAN	<0.001
**Perceptual Load**	UNREAL TOURNAMENT	PONG	0.0301
**Perceptual Load**	UNREAL TOURNAMENT	SIMS	<0.001
**Motor Load**	PAC-MAN	CALL OF DUTY	0.0348
**Motor Load**	SIMS	CALL OF DUTY	0.0004
**Motor Load**	PAC-MAN	MEDAL OF HONOR	0.0036
**Motor Load**	SIMS	MEDAL OF HONOR	<0.001
**Motor Load**	TETRIS	MEDAL OF HONOR	0.0401
**Motor Load**	UNREAL TOURNAMENT	PAC-MAN	0.0001
**Motor Load**	UNREAL TOURNAMENT	PONG	0.0348
**Motor Load**	UNREAL TOURNAMENT	SIMS	<0.001
**Motor Load**	UNREAL TOURNAMENT	TETRIS	0.0030

**Working Memory**	PAC-MAN	CALL OF DUTY	0.0301
**Working Memory**	PONG	CALL OF DUTY	0.0036
**Working Memory**	SIMS	CALL OF DUTY	0.0036
**Working Memory**	TETRIS	PAC-MAN	0.0401
**Working Memory**	UNREAL TOURNAMENT	PAC-MAN	0.0072
**Working Memory**	TETRIS	PONG	0.0051
**Working Memory**	UNREAL TOURNAMENT	PONG	0.0006
**Working Memory**	TETRIS	SIMS	0.0051
**Working Memory**	UNREAL TOURNAMENT	SIMS	0.0006
**Planification**	PAC-MAN	MEDAL OF HONOR	0.0119
**Planification**	SIMS	MEDAL OF HONOR	<0.001
**Planification**	TETRIS	PAC-MAN	0.0140
**Planification**	UNREAL TOURNAMENT	PAC-MAN	0.0030
**Planification**	TETRIS	SIMS	<0.001
**Planification**	UNREAL TOURNAMENT	SIMS	<0.001
**Target Accuracy**	PAC-MAN	CALL OF DUTY	0.0025

**Target Accuracy**	SIMS	CALL OF DUTY	<0.001
**Target Accuracy**	TETRIS	CALL OF DUTY	0.0164
**Target Accuracy**	PAC-MAN	MEDAL OF HONOR	0.0014
**Target Accuracy**	PONG	MEDAL OF HONOR	0.0461
**Target Accuracy**	SIMS	MEDAL OF HONOR	<0.001
**Target Accuracy**	TETRIS	MEDAL OF HONOR	0.0101
**Target Accuracy**	UNREAL TOURNAMENT	PAC-MAN	0.0001
**Target Accuracy**	UNREAL TOURNAMENT	PONG	0.0072
**Target Accuracy**	UNREAL TOURNAMENT	SIMS	<0.001
**Target Accuracy**	UNREAL TOURNAMENT	TETRIS	0.0012
**Divided Attention**	PAC-MAN	CALL OF DUTY	0.0119
**Divided Attention**	SIMS	CALL OF DUTY	<0.001
**Divided Attention**	TETRIS	CALL OF DUTY	0.0164
**Divided Attention**	PAC-MAN	MEDAL OF HONOR	0.0260

**Divided Attention**	SIMS	MEDAL OF HONOR	<0.001
**Divided Attention**	TETRIS	MEDAL OF HONOR	0.0348
**Divided Attention**	UNREAL TOURNAMENT	PAC-MAN	0.0086
**Divided Attention**	UNREAL TOURNAMENT	SIMS	<0.001
**Divided Attention**	UNREAL TOURNAMENT	TETRIS	0.0119
**Distraction Level**	PAC-MAN	CALL OF DUTY	0.0072
**Distraction Level**	SIMS	CALL OF DUTY	<0.001
**Distraction Level**	TETRIS	CALL OF DUTY	0.0003
**Distraction Level**	PAC-MAN	MEDAL OF HONOR	0.0119
**Distraction Level**	SIMS	MEDAL OF HONOR	<0.001
**Distraction Level**	TETRIS	MEDAL OF HONOR	0.0005
**Distraction Level**	UNREAL TOURNAMENT	PAC-MAN	0.0014
**Distraction Level**	UNREAL TOURNAMENT	SIMS	<0.001

**Distraction Level**	UNREAL TOURNAMENT	TETRIS	<0.001
**Level Progression**	SIMS	CALL OF DUTY	0.0008
**Level Progression**	TETRIS	MEDAL OF HONOR	0.0010
**Level Progression**	SIMS	PAC-MAN	0.0461
**Level Progression**	TETRIS	PAC-MAN	0.0051
**Level Progression**	SIMS	PONG	<0.001
**Level Progression**	TETRIS	SIMS	<0.001
**Level Progression**	UNREAL TOURNAMENT	TETRIS	0.0030

Table S8. Friedman test results by video game (within-game comparison across cognitive dimensions).

For each video game, the chi-squared statistic and associated p-value are reported from the Friedman test applied to expert ratings across all cognitive and perceptual dimensions. A p-value < 0.05 indicates that at least one dimension is significantly different from the others within that game.

**Video Game**	**Chi-squared**	**p-value**
Call of Duty	57.18	< 0.001
Medal of Honor	74.38	< 0.001
Unreal Tournament	76.96	< 0.001
Tetris	70.47	< 0.001
Pong	39.31	< 0.001
Pac-Man	21.16	0.0067
Sims	32.22	< 0.001

Table S9. Significant pairwise comparisons between cognitive dimensions within each video game (Nemenyi post hoc test).

For each video game, pairs of cognitive dimensions showing statistically significant differences ($p < 0.05$) are listed based on the Nemenyi post hoc test. Only significant comparisons are shown. R: Rhythm; PL: Perceptive Load; ML: Motor Load; WM: Working Memory; P: Planification; TA: Target Accuracy; DA: Divided Attention; D: Distraction; LP: Level Progression.

Video Game	Dimension 1	Dimension 2	p-value
Call of Duty	LP	PL	0.00085
Call of Duty	LP	DA	0.00253
Call of Duty	P	PL	0.00019
Call of Duty	P	DA	0.00062
Call of Duty	TA	LP	0.041
Call of Duty	WM	DA	0.029
Call of Duty	WM	PL	0.012

Video Game	Dimension 1	Dimension 2	p-value
Medal of Honor	LP	PL	7.87e-06
Medal of Honor	LP	DA	0.00011
Medal of Honor	WM	DA	0.00016
Medal of Honor	WM	LP	0.0046
Medal of Honor	WM	PL	1.17e-05
Medal of Honor	TA	LP	0.0012
Medal of Honor	PL	DA	0.00011
Medal of Honor	PL	LP	7.87e-06
Unreal Tournament	LP	DA	0.00016
Unreal Tournament	LP	ML	0.00016

Video Game	Dimension 1	Dimension 2	p-value
Unreal Tournament	LP	PL	0.00016
Unreal Tournament	WM	DA	0.041
Unreal Tournament	WM	ML	0.041
Unreal Tournament	WM	PL	0.041
Unreal Tournament	WM	R	0.041
Unreal Tournament	WM	TA	0.041
Tetris	LP	DA	0.0010
Tetris	P	D	4.24e-07
Tetris	PL	D	8.52e-04
Pong	WM	DA	0.012

Video Game	Dimension 1	Dimension 2	p-value
Pong	WM	ML	0.0039
Pong	WM	LP	0.00013
Pong	WM	TA	0.00099

S10 Table. Summary statistics (min, quartiles, mean, max) for each cognitive and perceptual dimension across all video games. Values represent the minimum, maximum, median, mean, and quartiles for each dimension

Variable	Min	Q1 (25%)	Median	Mean	Q3 (75%)	Max
**Rhythm of scenes**	1	3.00	4	3.40	4.00	5
**Perceptual load**	1	2.00	4	3.45	5.00	5
**Motor load**	1	2.00	4	3.38	4.00	5
**Working memory**	1	2.00	3	2.70	4.00	5
**Planification**	1	3.00	4	3.44	4.00	5
**Target accuracy**	1	2.00	3	3.31	4.25	5
**Divided attention**	1	2.00	4	3.48	5.00	5
**Level of distraction**	1	1.75	3	3.01	4.00	5
**Level load**	1	2.00	3	2.98	4.00	5

S11 Table. Average Euclidean distance between clusters (2D)

Cluster Comparison	Mean Euclidean Distance
1 vs 2	5.51
1 vs 3	3.21
2 vs 3	3.40

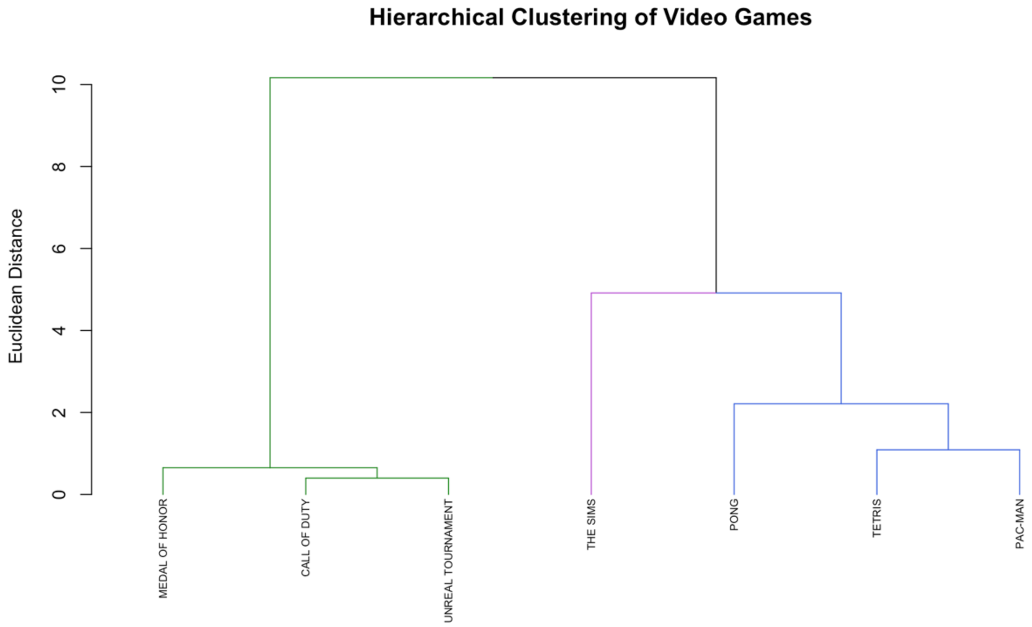

S12 Fig. Dendrogram of Hierarchical Clustering (Ward's Method). Cluster 1 is shown in blue, Cluster 2 in green, and Cluster 3 in purple.

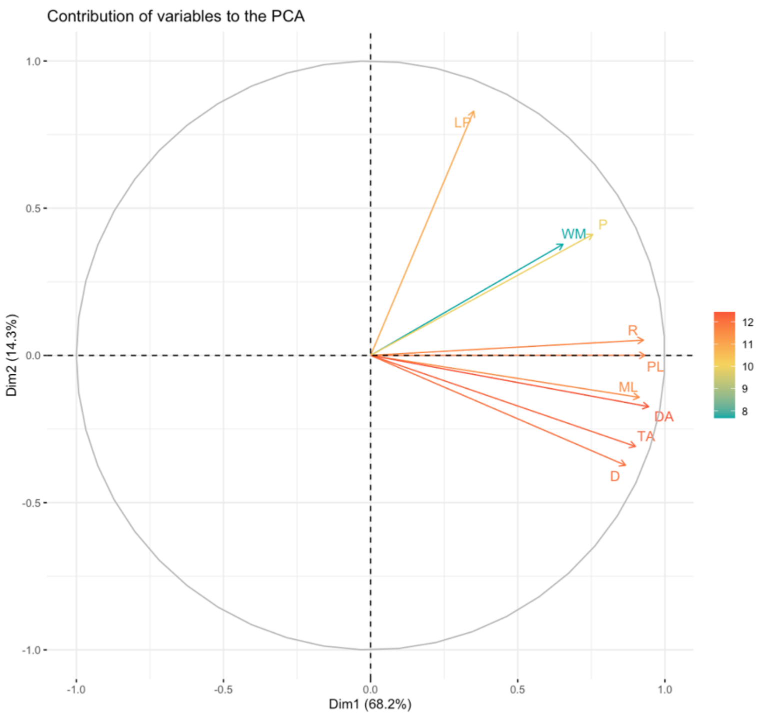

S13 Fig. Percentage of variance explained by each principal component in the PCA. The bar plot shows the proportion of total data variance captured by each component. Dim1, Dimension 1; Dim2, Dimension 2; R, Rhythm; PL, Perceptual Load; ML, Motor Load; WM, Working memory; P, Planification; TA, Target Accuracy; DA, Divided Attention; D, Distraction; Level Progression.

S14 Table. Percentage of variance explained by each PCA component.

Principal Component	Explained Variance (%)	Cumulative Variance (%)
**PC1**	68.18	68.18
**PC2**	14.31	82.50
**PC3**	6.76	89.25
**PC4**	3.92	93.17
**PC5**	1.97	95.14
**PC6**	1.64	96.78
**PC7**	1.34	98.13
**PC8**	1.06	99.19
**PC9**	0.81	100.00

S15 Table. Loadings of each original variable on the first five components.

Variable	PC1	PC2	PC3	PC4	PC5
**Rhythm of scenes**	-0.374	0.272	-0.046	-0.079	0.443
**Perceptual load**	-0.377	-0.032	-0.0003	-0.039	-0.581
**Motor load**	-0.369	0.218	0.126	0.232	0.465
**Working memory**	-0.264	-0.783	-0.333	-0.352	0.256
**Planification**	-0.305	-0.180	-0.362	0.802	-0.129
**Target accuracy**	-0.363	0.017	0.272	-0.079	-0.299
**Divided attention**	-0.381	0.092	0.153	-0.128	0.129
**Level of distraction**	-0.350	-0.047	0.329	-0.192	-0.182
**Level load**	-0.141	0.470	-0.731	-0.335	-0.167

S16 Table. MDS 2D coordinates for each game

VideoGame	Visuomotor	Cognitive	Cluster
**CALL OF DUTY**	-2.9314340	0.32559510	1
**MEDAL OF HONOR**	-2.8901892	-0.86013289	1
**PAC-MAN**	2.6366117	0.08249683	3
**PONG**	1.0597265	-0.25804190	3
**SIMS**	5.3752200	-1.36885489	2
**TETRIS**	0.5547595	2.78406654	3
**UNREAL TOURNAMENT**	-3.8046945	-0.70512879	1

S17 Table. MDS 3D coordinates for each game

VideoGame	Visuomotor	Cognitive	AttentionalControl
**CALL OF DUTY**	-2.9728277	-0.02478757	0.53596875
**MEDAL OF HONOR**	-3.0415999	0.81349918	-0.13714300
**PAC-MAN**	2.5788317	-0.07172331	-0.30688823
**PONG**	1.0683333	-0.20685342	-1.11116286
**The Sims**	5.2678527	1.41827075	0.49141913
**TETRIS**	0.8161089	-2.66331765	0.45590128
**UNREAL TOURNAMENT**	-3.7166990	0.73491201	0.07190495

S18 Table. Euclidean distance matrix

	Call of Duty	Medal of Honor	Pac-Man	Pong	Sims	Tetris	Unreal Tournament
**Call of Duty**	0.000	1.349	5.595	4.140	8.332	4.830	1.512
**Medal of Honor**	1.349	0.000	5.625	4.082	8.302	5.047	1.244
**Pac-Man**	5.595	5.625	0.000	2.009	3.309	3.429	6.454
**Pong**	4.140	4.082	2.009	0.000	4.992	2.771	5.006
**Sims**	8.332	8.302	3.309	4.992	0.000	6.060	9.178
**Tetris**	4.830	5.047	3.429	2.771	6.060	0.000	5.613
**Unreal Tournament**	1.512	1.244	6.454	5.006	9.178	5.613	0.000

S19 Table. Mean global rating per video game and expert.

	EXPERT												
**VIDEO GAME**	**1**	**2**	**3**	**4**	**5**	**6**	**7**	**8**	**9**	**10**	**11**	**12**	**Total**
**Call of duty**	4.22	4.00	4.11	3.89	4.33	4.22	4.11	4.22	4.11	4.11	4.22	3.89	4.12
**Medal of honor**	4.33	4.22	4.11	3.89	4.56	4.22	3.67	4.22	3.89	4.00	4.11	3.78	4.08
**Unreal tournament**	4.33	4.33	4.67	4.56	4.56	3.78	4.44	4.67	4.00	4.56	4.33	4.67	4.41
**Tetris**	3.11	3.22	2.67	3.22	2.89	3.00	3.33	3.44	2.67	3.78	3.33	3.78	3.20
**Pong**	2.44	2.33	3.11	3.13	3.00	3.67	2.56	2.67	3.33	3.33	2.33	3.67	2.97
**Pac-man**	2.00	2.22	1.78	2.78	2.33	3.22	2.22	2.33	2.56	2.33	2.22	3.11	2.43
**Sims**	1.11	1.11	1.56	1.11	1.11	1.78	2.00	1.22	1.33	1.67	1.33	1.89	1.44

S20 Table. Pairwise Wilcoxon signed-rank tests between video games.

P-values		**VIDEOGAMES**						
		**Call of duty**	**Medal of honor**	**Unreal tournament**	**Tetris**	**Pong**	**Pac-man**	**Sims**
**VIDEOGAMES**	**Call of duty**	-	*0.725*	*0.020*	*0.003*	*0.003*	*<0.001*	*0.003*
	**Medal of honor**	*0.725*	-	*0.019*	*0.004*	*0.003*	*0.003*	*0.003*
	**Unreal tournament**	*0.020*	*0.019*	-	*0.003*	*0.002*	*0.003*	*0.003*
	**Tetris**	*0.003*	*0.004*	*0.003*	-	*0.142*	*0.004*	*0.003*
	**Pong**	*0.003*	*0.003*	*0.002*	*0.142*	-	*0.003*	*0.003*
	**Pac-man**	*<0.001*	*0.003*	*0.003*	*0.004*	*0.003*	-	*0.003*
	**Sims**	*0.003*	*0.003*	*0.003*	*0.003*	*0.003*	*0.003*	-
